# The Clinical and Molecular Features in the VHL Renal Cancers; Close or Distant Relatives with Sporadic Clear Cell Renal Cell Carcinoma?

**DOI:** 10.3390/cancers14215352

**Published:** 2022-10-30

**Authors:** Alessandra Cinque, Roberto Minnei, Matteo Floris, Francesco Trevisani

**Affiliations:** 1 Biorek S.r.l., San Raffaele Scientific Institute, 20132 Milan, Italy; 2 Nephrology, Dialysis, and Transplantation, G. Brotzu Hospital, University of Cagliari, 09134 Cagliari, Italy; 3 Urological Research Institute, San Raffaele Scientific Institute, 20132 Milan, Italy; 4 Unit of Urology, San Raffaele Scientific Institute, 20132 Milan, Italy

**Keywords:** VHL, clear cell renal cell carcinoma (ccRCC), miRNA, biomarkers, von Hippel Lindau syndrome, sporadic ccRCC

## Abstract

**Simple Summary:**

VHL syndrome is an autosomal dominant hereditary cancer syndrome and one of the leading causes of death is ccRCC. Current target therapies may lead to stable disease or partial remission, as best response and scant evidence supports the therapeutic decision-making in metastatic ccRCC. Therefore, new genetic and epigenetics insights are needed, to guide the development of more effective target therapies and to promptly predict the presence and prognosis of ccRCC. Our review highlights all the new molecular perspectives based on research of genetic alterations, biological pathways and promising biomarkers in the VHL-associated hereditary ccRCC.

**Abstract:**

Von Hippel-Lindau (VHL) disease is an autosomal dominant inherited cancer syndrome caused by germline mutations in the VHL tumor suppressor gene, characterized by the susceptibility to a wide array of benign and malign neoplasms, including clear-cell renal cell carcinoma. Moreover, VHL somatic inactivation is a crucial molecular event also in sporadic ccRCCs tumorigenesis. While systemic biomarkers in the VHL syndrome do not currently play a role in clinical practice, a new promising class of predictive biomarkers, microRNAs, has been increasingly studied. Lots of pan-genomic studies have deeply investigated the possible biological role of microRNAs in the development and progression of sporadic ccRCC; however, few studies have investigated the miRNA profile in VHL patients. Our review summarize all the new insights related to clinical and molecular features in VHL renal cancers, with a particular focus on the overlap with sporadic ccRCC.

## 1. Epidemiology, Clinical Features, Diagnosis and Screening of VHL Disease

### 1.1. Current Epidemiology and Clinical Spectrum

VHL disease is a rare inherited cancer syndrome, with a birth incidence between 1/27,300 and 1/45,500 live births, and a prevalence rate ranging between 1/39,000 and 1/91,000 individuals [1]. VHL-related tumors are usually diagnosed in adult age, although an earlier age of onset can be observed [2] depending on the VHL disease type and presenting clinical manifestations [3]. The most frequently reported presenting manifestation is central nervous system hemangioblastoma (CHB), followed by retinal hemangioblastoma (RHB), RCC, pancreatic cystic lesions, pheochromocytoma (Pheo), and genital system lesions (GSLs). CHB, RHB, and GSLs are associated with earlier clinical onset, at 29.5, 24.8, and 12.4 years of age, respectively [4].

### 1.2. VHL Disease Types, Clinical Spectrum

Von Hippel in 1904 and Lindau in 1927 were among the first to describe some features of VHL disease (retinal angiomatosis and cerebellar-spinal hemangioblastomas, respectively) [5,6]. VHL disease is characterized by a susceptibility to a wide array of tumors, including CHB (with cerebellar and spinal localization) and RHB, Pheo, endolymphatic sac tumors (ELSTs) of the middle ear, neuroendocrine tumors and serous cystadenomas of the pancreas, GSLs (such as papillary cystadenomas of the broad ligament and the epididymis) and, lastly, hereditary ccRCCs [7], with the latter being one of the main causes of death in this population, the leading one according to some studies [7,8,9].

VHL disease has diverse genotype and phenotype correlations, and variable intra- and inter-familial expressivity [10,11,12].

VHL disease can be classically divided into various types and subtypes according to the presence or absence of Pheo and ccRCC susceptibility: type 1 is the most common, it often displays deletions or truncating mutations and does not predispose to the development of Pheo; on the contrary, type 2 disease (7–20% of families) exhibits missense mutations and Pheo susceptibility [13,14,15]. Type 2 VHL disease can be further classified as type 2A (not including ccRCC), type 2C (Pheo is the only disease manifestation), and type 2B (featuring both ccRCCs and Pheo) [15].

**Figure 1:**
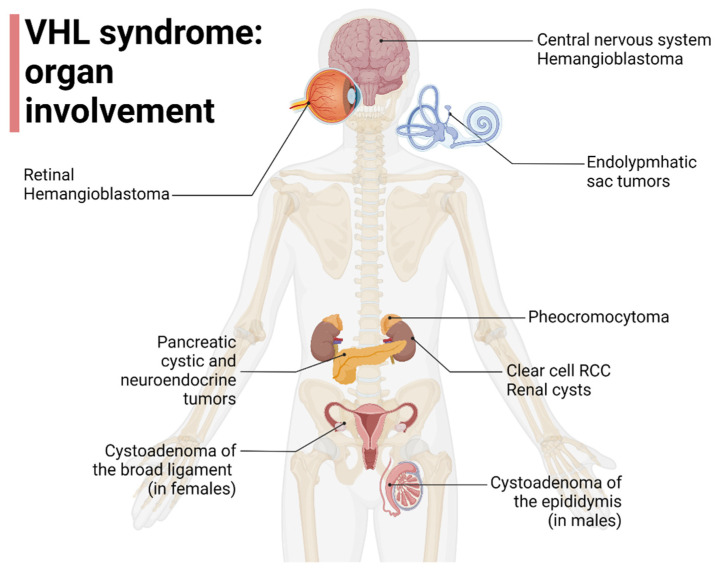
Organ involvement in VHL disease, adapted from “Human Internal organs”, by BioRender.com (2022). Retrieved from https://app.biorender.com/biorender-templates (accessed on 1 October 2022).

Since affected families can shift between subtypes over time, the clinical utility of this classification is limited [15]. Liu et al. reported that VHL gene mutations affecting HIF1a binding (HM and TR) can increase the risk of developing ccRCC [16], furthermore, Gallou et al. found that mutations in the missense cluster regions were associated with an increased risk of RCC compared to those with missense mutations in other regions [17]. Kai R et al. and Liu et al. found, instead, that missense mutations in MCR are not associated with RCC risk, suggesting the possible contribution of a different mechanism from those involving VHL-HIF [16,18]. The organ involvement in VHL disease is represented in Figure 1.

### 1.3. Diagnostic Criteria and Screening

First diagnostic criteria—1964: patients with no family history of VHL disease and two related tumors (two CHBs alone or only one combined with a visceral tumor), or with family history and at least one VHL-related tumor [19]. Clinical and genetic findings are included in the current diagnostic criteria, including CHB, RHB, RCC, Pheo, paraganglioma, glomus tumor, ELST, pancreatic neuroendocrine tumors, and/or cysts. Even without a CHB or RHB, VHL disease can be diagnosed with at least one of the aforementioned manifestations and a positive VHL genetic test or with positive family history for VHL in at least one first-degree relative [20].

Careful family history and screening for other VHL-related tumors, in all patients who undergo hemangioblastoma genetic testing for VHL disease, is recommended [21].

Genetic testing on peripheral blood samples should be offered to all clinically suspected cases. Southern blotting (SB) is usually used to detect whole gene deletion and gene rearrangements, and fluorescence in situ hybridization has a consequent confirmatory role, but multiplex ligation-dependent probe amplification can be alternatively used instead of SB. In patients with a negative result on peripheral blood, genetic testing can be repeated by sampling other tissues [20].

In established cases, a comprehensive screening program is recommended, while with regard to ccRCC, an annual abdominal MRI starting from the ages of 10–16 years of age is of paramount importance. An annual abdominal ultrasound is a complementary technique, while abdominal CT is reserved for clinical scenarios in which MRI is contraindicated [2,22,23]. Screening programs vary across institutions, for example, Maher et al. proposed an annual ophthalmic examination for hemangioblastoma (starting from the age of 1), contrast-enhanced MRI of the brain and spine for hemangioblastomas of the central nervous system (starting at the age of 12), annual blood pressure evaluation and 24 h urinalysis for catecholamines and their metabolites or plasma free metanephrines for Pheo (starting at the age of 4 years), audiogram every two years for endolymphatic sac tumors (starting at the age of 16 years) and, finally, annual MRI of the abdomen for RCCs and pancreatic tumors (starting at the age of 12 years) [22].

## 2. Molecular Features of VHL Disease

### 2.1. Background and Overview of VHL Disease Genetics

In VHL disease, tumor susceptibility is underlain by genetic abnormalities behaving as tumor suppressor genes, causing a dysfunctional VHL-Hypoxia Inducible Factor (HIF) pathway, according to the 2-hit hypothesis for the development of cancer and Knudson’s theory of human carcinogenesis [24,25]. The first event is the inheritance of a germline pathogenic gene variant that results in a loss of function of one allele of the VHL-elongin C and elongin B (VCB) complex in all cells. This predisposing condition has to be followed by a second somatic event that, genetically or epigenetically, inactivates the wild-type allele in the cells involved by this acquired insult, finally leading to a loss of function of the VCB complex [24,26]. The inactivation of both alleles is crucial in carcinogenesis [27], and reinstatement of VHL function can partially reverse some effects of the biallelic inactivation [28]. High penetrance is observed, and up to 90% of patients will exhibit VHL-related clinical manifestations [1,7,15].

However, in about 20% of affected families, no VHL deletion or mutation is detectable [13]. VHL disease is associated with different molecular abnormalities besides the loss of the VHL gene, such as the loss of segments of the small arm of chromosome 3 (the VHL gene-harboring 3p25-26 [27], 3p12, 3p13-14.2, 3p21) [29], and several novel mutations involving elongin C-VHL binding (TCEB1 gene), BAP1 and SETD2 genes and several pathways (PI3K-AKT-mTOR, DNA methylation, p53-related and mRNA processing) [30,31].

Notably, VHL loss of function is not sufficient for ccRCCs to develop, [32,33] since this genetic anomaly is already present in benign renal cysts without foci of ccRCCs in VHL-deficient patients, as a consequence, mechanisms other than the VHL-HIF pathway are most likely involved in RCC progression [32,34].

**Figure 2:**
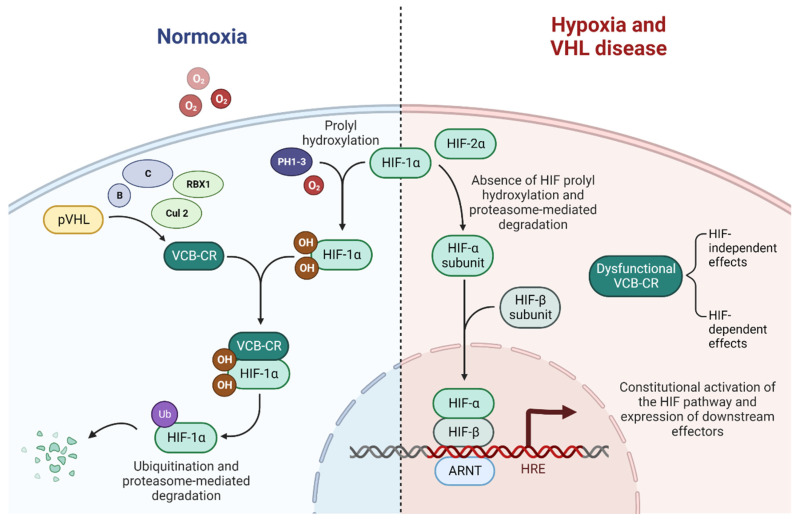
The VHL-HIF pathway in normoxia, hypoxia, and VHL disease, adapted from “HIF signaling”, by BioRender.com (2022). Retrieved from https://app.biorender.com/biorender-templates (accessed on 1 October 2022).

A disrupted VHL pathway leads to dysfunctional ubiquitination of the VCB-Cullin 2-RBX1 (VCB-CR) complex and consequently to the constitutional activation of the HIF pathway and, finally, to subsequent alterations in downstream processes, mediated by abnormally expressed carcinogenic factors [35,36,37]. Alongside the HIF-related effect, several HIF-independent effects result from the loss of function of the VHL pathway [38]. A schematic of the VHL-HIF pathway is presented in Figure 2.

### 2.2. VHL Gene, pVHL, HIF, and HIF-Independent Functions of pVHL

The VHL locus was first cloned and mapped between the late 80s and early 90s, while the VHL gene was identified in 1993 [39,40]. The coding sequence includes three exons, and based on the presence of exon 2, there are two mRNA isoforms [13]. The gene product is the VHL protein (pVHL), which consists of two isoforms, both of which have tumor-suppressing activity [41].

pVHL binds elongin C and elongin B to form the VCB complex [42], stabilizing its component and sheltering them from proteasomal degradation [43], and conferring a ubiquitin ligase activity, acting as an E3 ligase [34,44,45]. The VCB complex then binds to cullin 2 and RBX1 to form the VCB-CR complex [46].

In VHL-wild type patients in normoxic conditions, the VCB-CR complex is able to bind to prolyl hydroxylated hypoxia-induced factor 1α (HIF1α), while with hypoxia, this binding is prevented by the lack of hydroxylation of HIF1α by prolyl hydroxylase 1, 2 and 3, inactivated by the lack of their co-substrate: oxygen [47,48].

In VHL disease, the loss of function in the VCB-CR complex prevents its binding to the ubiquitous unstable subunit HIF1α, which subsequently heterodimerizes with the stable HIF1β to form HIF1, which translocates to the nucleus and acts as a transcription factor binding the hypoxia-response elements, leading to the constitutive activation of expressed downstream effectors that would physiologically be activated only in hypoxic conditions. HIF is a key driver of ccRCC progression and might be a tumor suppressor, directly contributing to tumorigenesis [34,49,50].

Some of the most relevant downstream effectors are TGFα, EGFR (involved in cell proliferation and survival), PDGFB (angiogenesis, lymphangiogenesis), the chemokine receptor CXCR4 and MMP2/9/14 and lysyl oxidase, UPAR, MMP2 (tumoral cell invasion and metastases), dysregulation of TWIST and activation of HGFR (epithelial-to-mesenchymal transition), VEGFA (angiogenesis) [50,51].

HIF2α is a subunit isoform peculiarly expressed only in the endothelium, lung, liver, and kidney cells, activated in hypoxia, that can heterodimerize with a β-subunit (HIF1β) to form HIF2, which displays a slightly different spectrum of downstream effects compared to HIF1, preferentially driving growth and angiogenetic processes [52]. HIF2, more than HIF1, appears to be a more prominent driver of RCC tumorigenesis [52,53,54]. It is known to activate mTOR complex 1 [55,56] and is increasingly being investigated as a target for direct inhibition in VHL-deficient ccRCCs [57,58]. Belzutifan, an HIF-2a inhibitor, was approved by the Food and Drug Administration on 13 August 2021 for the treatment of RCCs, CHB, and pancreatic neuroendocrine tumors not immediately requiring surgery [59].

**Figure 3:**
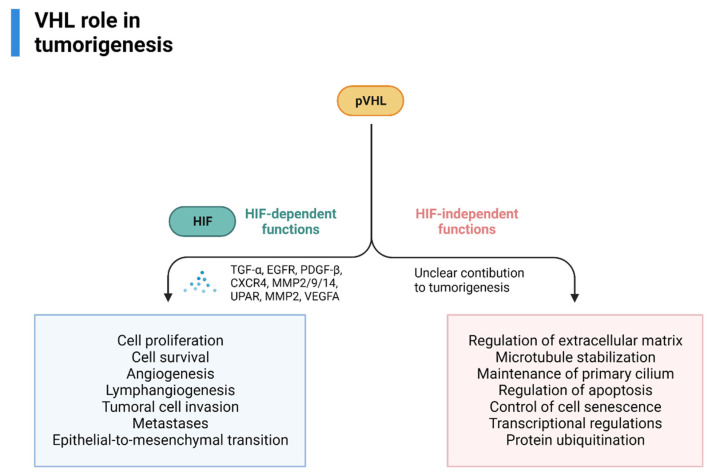
VHL role in VHL-related RCC tumorigenesis, adapted from “Cell Differentiation Pathway (Layout, Vertical) 2”, by BioRender.com (2022). Retrieved from https://app.biorender.com/biorender-templates (accessed on 1 October 2022).

pVHL also has HIF-independent functions (assembly and regulation of extracellular matrix, microtubule stabilization, maintenance of primary cilium, regulation of apoptosis, control of cell senescence-survival, transcriptional regulations, protein ubiquitination) that could, theoretically, influence tumorigenesis, however, its contribution is currently unknown [60,61,62,63]. An overview of VHL’s role in RCC tumorigenesis is provided in Figure 3.

### 2.3. Difference between VHL-Related Sporadic and Hereditary ccRCC

The genetic abnormalities in sporadic ccRCC are similar to those observed in VHL disease, i.e., loss of 3p25, and VHL mutations are present in 60–90% of patients [64].

VHL mutations are typically frameshift or nonsense mutations, but missense mutations may be observed [65]. Epigenetic abnormalities have been described, and in particular, VHL silencing by methylation is present in 5–30% of sporadic ccRCCs, thus resulting in LOH in up to 98% of cases [66]. Biallelic inactivation of the VHL gene in sporadic ccRCCs is lower than expected, and Hamano et al. reported that only 44% of LOH ccRCCs showed this feature, implying the existence of a different 2nd hit mutational event [67].

Sporadic ccRCCs display mutation heterogeneity, with different subclones harboring different anomalies. In progressively smaller subclones, genetic abnormalities are named ubiquitous (such as VHL loss and chromosome 3p loss, early, truncal events, in every tumor cell), shared, and private [68].

VHL mutational status does not predict oncologic outcomes in sporadic ccRCCs since mixed results have been reported. Masahiro et al. reported a strong association between VHL alterations in sporadic ccRCC and better cancer-free survival and cancer-specific survival in non-metastatic disease treated with radical nephrectomy (RN), particularly in stage III patients [69]. On the contrary, Jung Han et al. could not find an association between somatic VHL alterations and prognosis in sporadic ccRCC, although patients with loss-of-function VHL mutations had decreased progression-free survival and overall survival [70]. Inactivation of the VHL gene in sporadic ccRCC is not associated with nuclear grade, disease stage, or overall survival, according to a recent meta-analysis by Kim et al. [71].

Gordan et al. reported an alternative classification of sporadic ccRCC based on the VHL allele, HIF1a, HIF2a, and MYC expression, recognizing three different molecular subgroups: VHL WT (wild-type VHL alleles, undetectable HIFα protein), H1H2 (VHL-deficient, expressing HIF1α and HIF2α proteins) and H2 tumors (VHL-deficient, expressing only HIF2α, enhanced MYC activity, higher proliferation rates) [72].

### 2.4. Clinical and Therapeutic Implications, Prognosis

The prognosis could be improved by performing a genetic diagnosis and careful follow-up [73]. Before the introduction of surveillance protocols, life expectancy was less than 50 years [13], but by adopting a regular screening program, earlier diagnosis and treatment of VHL-related tumors led to an extension of life expectancy to between 52.5 and 66 years [4,74,75].

Especially in advanced diseases, the prognosis remains poor. The incomplete understanding of VHL tumorigenesis slowed down the development of a rich target-therapy arsenal [38]. However, several steps forward have been made in understanding its pathogenesis, tailoring the surgical strategy, and enriching the arsenal of antineoplastic drugs with valid target-directed agents and immune-checkpoint inhibitors [35].

A poor prognosis is associated with advanced-stage disease, which implies a limited response to chemotherapy and radiotherapy, but a better response to immunotherapy, biologics, or target therapy [76].

Early-onset disease, a positive family history, and truncating VHL mutations are associated with reduced overall survival and disease-specific survival [75], and female sex is associated with decreased overall survival [77].

Risk factors for overall survival in VHL disease also include truncating mutations, type 1 disease, presence of CNB or RHB rather than abdominal lesions (RCCs, pancreatic neoplasms, Pheo) [4].

Several molecular pathways are involved in tumorigenesis and, consequently were investigated as possible therapeutic targets in this population, especially multiple tyrosine kinase inhibitors (TKis) linked to key downstream effectors. However, it was only recently that another class of drugs proved to be active and well tolerated in VHL patients, leading to FDA approval of the HIF-2a inhibitor Belzutifan [59].

## 3. Von Hippel-Lindau Disease and ccRCC

### 3.1. Clinical, Pathological, and Imaging Overview

ccRCC is acknowledged as a Renal Cell Tumor by 2016 WHO Classification of kidney tumors [78], representing the most common RCC histotype and accounting for 70% of cases [79].

VHL syndrome is the oldest and most common hereditary RCC syndrome, among at least twelve known entities [76].

Hereditary cancer syndrome of the kidney represents 5–8% of cases [80]. In a study by Ferstl et al., the prevalence of VHL RCC was 1.6% in a series of unselected RCCs [81].

VHL disease predisposes to the development of renal cysts and ccRCCs, and a single individual can present with multiple lesions [82]. A nationwide audit reported a median of 2–3 RCCs per individual [83,84]. In renal cysts, foci of dysplasia or RCC can be present, especially in complex cysts [82,84]. Foci of RCC can also be seen in the context of simple renal cysts [85], and VHL deficiency can also be observed in the cystic epithelium, supporting the hypothesis of cysts as precursor lesions of ccRCC in VHL disease [86].

ccRCC will develop in 30–70% of patients with VHL-disease [7,13,23,87]. The mean age at presentation is 39–44 years, the disease rarely presents before 20 years of age [37] but can occur between 15 and 75 years [73]. The probability of developing ccRCC by the age of 60 years is 0.69. Before the introduction of modern screening protocols, the reported median survival was 49 years and ccRCC was the leading cause of death [7,88]. VHL- and RCC-related deaths have decreased over time, with hemangioblastoma representing the first cause of death in this population [75].

In VHL patients with ccRCC, the 10-year disease-specific survival rate is 95% [73]. The overall survival in VHL-deficient individuals is progressively approaching that of unaffected siblings and of the general population [75].

Hereditary VHL-related ccRCCs display earlier onset and multifocal and bilateral tumors [88]: Neumann et al. described age at onset as being 25 years less than in sporadic ccRCCs, and an association with renal cyst and cystic organization, low histological grade, absence of metastasis in tumors smaller than 7 cm and better 10-year survival [81]. Poston et al. also documented that ccRCC in VHL disease tends to have a lower grade, with low local invasiveness [84]. Other authors, on the other hand, have reported a similar growth rate between sporadic and VHL disease ccRCCs [82,89,90].

The metastatic rate is around 11%, lower than for sporadic ccRCCs [73], and it is highly influenced by the tumor size and growth rate [91].

VHL disease displays a high rate of recurrence [76]; Ploussard et al. documented that among patients who underwent nephron-sparing surgery, 45.6% and 83.7% experienced a local recurrence at 5 and 10 years, respectively, with a 5-year and 10-year overall repeat surgery rate of 21.1% and 63.4%, respectively [92].

Diagnosis is often delayed due to the asymptomatic nature of the tumors, thus typical signs and symptoms of RCC, such as visible hematuria, flank pain, and palpable flank mass or masses are indicative of more advanced disease [88].

Unlike in the screening setting, the first-line diagnostic technique in a suspected case of RCC is contrast-enhanced abdominal CT to characterize the location, number, size, and appearance of solid lesions [88].

The predominant histotype in VHL disease is ccRCC. A series on renal pathology in VHL disease showed that 91% of solid neoplasms in surgical specimens were ccRCCs, with different cytological features (pure clear cell features, clear cells with dispersed granular cells, and sarcomatoid RCC containing clear and granular cells in 53%, 45% and 1.5% of cases, respectively). The architectural pattern was typically trabecular, with a minority of cases displaying a microcystic pattern. The renal masses were all well-circumscribed, and a pseudocapsule (PSC) frequently surrounded the neoplasm. In this series, PSC microscopic invasion was present in 76% of cases, with no invasion of the surrounding parenchyma [84]. The neoplastic cells are large and have abundant clear cytoplasm that is rich in glycogen and lipids [93]. Wild-type sporadic ccRCCs may have different histological and clinical features, in a 10-year retrospective study, wild-type ccRCCs were associated with a higher nuclear grade, sarcomatoid component, dense lymphocyte infiltrate, nodal involvement, and presence of metastases compared to VHL-inactivated ccRCCs patients, with a markedly reduced disease-specific survival of 33 months versus 107 months, respectively [94]. A recent retrospective study by Wang et al. evaluated clinicopathological predictive markers in VHL-associated ccRCCs and in sporadic ccRCCs. The authors reported a higher histologic grade in the sporadic group, which exhibited a faster growth rate [95]. A kidney biopsy is seldom needed in patients with established VHL disease. Chahoud et al. recently published a report on the evaluation and diagnosis of limited renal masses in VHL disease, stating that a kidney biopsy can be indicated in selected clinical settings, i.e., in case of atypical imaging features suggesting a benign lesion (infections, foci of inflammation), in case of a positive history of extra-renal malignancy that could disseminate to the kidney prior to percutaneous ablation, or when post-interventional recurrence is suspected [96].

US is not a sensitive enough technique to characterize RCCs and, when used alone, is outperformed by MRI and CT [97].

The Bosniak system classification is useful for describing cystic lesions in VHL disease, but since small foci of dysplasia or RCC can harbor in these lesions, it should not be used to guide management in this population of patients, unlike in sporadic ccRCCs [98].

A national audit of VHL disease showed that MRI was the most common detection modality, followed by CT and ultrasonography, especially for bigger lesions [83]. MRI is better at detecting small renal masses than ultrasonography [83] and, although its sensitivity is inferior to that of abdominal CT, its use is preferred in screening protocols to spare patients from cumulative exposure due to ionizing radiations starting at a young age [83,99].

**Figure 4:**
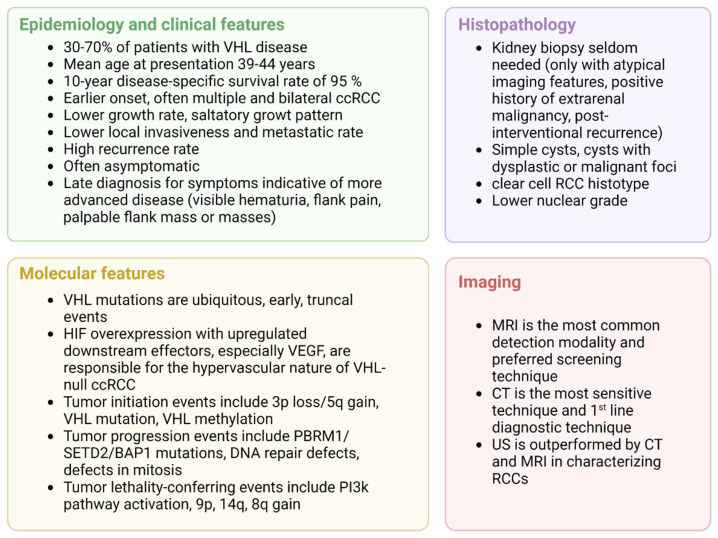
Clinical overview of VHL-related ccRCC, adapted from “4 Panels (Layout 2 × 2)”, by BioRender.com (2022). Retrieved from https://app.biorender.com/biorender-templates (accessed on 1 October 2022).

VHL has a saltatory growth pattern, with quiescent phases, that complicates the interpretation of imaging findings as treatment-response or natural quiescence [38]. A summary of the clinical key aspects of VHL-related ccRCC is presented in Figure 4.

### 3.2. Peculiar Molecular Aspects of VHL-Related ccRCC

VHL mutations are ubiquitous, early, truncal events in ccRCCs [68].

Jonasch et al. found an association between the molecular abnormalities underlying ccRCCs and their timing and subdivided them into those that drive tumor initiation events (3p loss/5q gain, VHL mutation, VHL methylation), tumor progression (PBRM1/SETD2/BAP1 mutations, DNA repair defects, defects in mitosis) and those that confer lethality (PI3K pathway activation, 9p, 14q, 8q gain) [100].

ccRCCs are hypervascular tumors, and the main driving factor in tumor progression is represented by HIF overexpression and upregulated downstream effectors, above all VEGF [12]. The endothelium of VHL-deficient patients is highly dysfunctional, displaying altered cell adhesion, angiogenesis, migration, immune response, cell metabolism, and ROS homeostasis [101].

ccRCCs typically display large neoplastic cells, featuring an abundant, clear cytoplasm rich in glycogen and lipids, derived from a high glucose metabolism that, in VHL-null ccRCCs, can possibly be linked to the increased expression of an HIF downstream effector (the adipose differentiation-related peptide) acting as a lipid transporter [93]. A schematic of the genetic alterations, biological pathways involved, and investigated biomarkers in VHL syndrome RCC is synthetized in Table 1 and Table 2.

## 4. Therapeutic Approach to VHL-Related ccRCC and Clinical Outcomes

### 4.1. Localized Disease

In the last decades, the implementation of genetic testing and the improvements in screening protocols have led to better clinical outcomes. The aim is to detect RCCs before the development of metastatic disease and to approach renal masses with nephron-sparing surgery so as to maximally preserve renal function. In view of the multifocal and bilateral disease presentation, the high recurrence rate, and the limited number of possible nephron-sparing surgical interventions because of their effects on renal function, tailored and careful surgical planning is of paramount importance.

Regarding target therapy, a recent phase II study of HIF-2a inhibitors in ccRCCs documented objective partial responses in 30 of the 61 enrolled patients, stable disease in 30 patients, and only one case of disease progression after a median follow-up time of 21.8 months [102]. The investigated drug, Belzutifan, proved to be active in renal and non-renal VHL-related cancer and well-tolerated by VHL patients, leading to its approval by the FDA on 13 August 2021 [59].

Lesions with a maximum diameter of less than 3 cm are usually treated with a surveillance protocol. The VHL alliance recommends repeating abdominal MRI every 3 to 6 months to assess tumor growth. In case of disease stability over 3 consecutive MRIs, the interval between scans can be extended to 2 years. On the contrary, if a lesion is greater than 3 cm, a urologic referral is mandatory [103]

Walther et al. investigated the 3 cm threshold as an indication for surgery; smaller tumors (group 1) underwent imaging surveillance and eventual surgery when the threshold was reached, while larger tumors (group 2) were immediately resected. After 60 months, no group 1 patients developed metastatic disease and only one required surgery, while after a 66-month follow-up, 12 of 44 group 2 patients required nephrectomy, and 11 of 44 progressed to the metastatic stage [104].

Surgery is the standard of care when dealing with >3 cm lesions, although the recent approval of Belzutifan in this setting has enriched the therapeutic arsenal and can be offered as an alternative choice. Belzutifan approval is based on the NCT03401788 trial, which included individuals with renal masses ranging from 10 to 61mm with no evidence of metastatic disease [102]. It is not currently approved for metastatic disease but can be offered to patients with <3 cm renal lesions. A summary of the drugs or compounds evaluated in a clinical trial for the treatment of VHL disease-ccRCC is provided in Table 3.

A maximum renal mass diameter greater than 3 cm is associated with an increased risk of metastatic disease, although VHL-related ccRCCs tend to have limited local invasiveness and histologic grade [88,105]. Radical nephrectomy (RN) is not the first choice since nephron-sparing surgery (NSS) is effective and preserves renal function.

Steinbach et al. reported similar 5- and 10-year disease-specific survival rates for RN (95% and 77%, respectively) and NSS (100% and 81%, respectively). Moreover, the NSS group had 5- and 10-year recurrence-free survival rates of 71% and 15%, 51% displayed postoperative local recurrence, and only 2 of 49 patients progressed to metastatic disease. Finally, 23% developed ESKD, 6 of 15 were managed with renal transplantation, and 9 with dialysis [106]. Bratslavsky et al. investigated the outcomes of salvage partial nephrectomy (PN), intended as a 3rd or 4th PN on the same operated kidney. Twenty-three% of patients lost the renal unit, while the rest avoided the need for dialysis over a 25-month median follow-up [107].

Some investigators suggested adopting a 4 cm threshold to guide the treatment of renal masses in VHL disease, in an effort to maximally preserve the quality of life, renal survival, and overall survival [108].

A nationwide Japanese survey found that 203 VHL disease patients were affected by RCC (50.3%). Partial nephrectomy was performed in 46% of cases, radical nephrectomy in 31%, radiofrequency ablation (RFA) in 14%, and in 44% of cases two or more interventions were necessary. An increased number of surgeries correlated with a decrease in eGFR. The 10-year cancer-specific survival rate was higher than in non-VHL disease patients, reaching 95% [73]. In a UK national audit of VHL disease, reporting real-world data, clinical outcome was reported for 229 patients with RCC, of whom 22.2%, 58.5%, and 19.3% underwent clinical monitoring, surgery, and RFA or CB, respectively. Over a 5-year period, only 1.8% of patients had subsequent end-stage kidney disease (mean age 40.5 years), and 46.6% underwent kidney transplantation (mean age 37.3 years) [83].

When NSS is not feasible or does not prevent ESKD, renal replacement therapy is usually needed. Goldfarb et al. specifically investigated the clinical outcomes in VHL patients who underwent RN, developed ESKD, and received a renal transplantation, and compared the data with a control group of non-VHL transplanted patients. They reported that graft survival, patient survival, and renal function were similar between the two groups, supporting the safety of RT in VHL disease [109].

Surgery is usually not necessary for smaller than 3cm lesions due to the low grade and low metastatic rates [110], while larger masses, which are often multifocal and bilateral, should be treated with a nephron-sparing strategy to reduce the risk of inducing chronic kidney disease. The 10-year survival rate of the latter group of patients is 81% [20]. After a mean of 60 months follow-up following nephron sparing surgery for <3 cm tumors, Walther et al. reported no metastatic events and none of the patients needed renal replacement therapy [104].

Although smaller lesions are usually managed by close surveillance or Belzutifan, ablative techniques such as percutaneous RFA are increasingly being investigated for the treatment of <3 cm lesions, because of their efficacy and low complication rates [111]. Cryoablation has the advantage of being less painful and can ablate larger masses but, on account of its impact on renal function and technical feasibility, RFA is preferable in VHL disease [110]. RFA is not performed on <1 cm lesions because of the difficult imaging characterization leading to false positive and false negative results, and due to the low metastatic risk [110]. The most appropriate settings for nephron-sparing surgery are in patients with small renal masses of 2–3 cm diameter, not close to vessels or the bowel, or if unsuitable for more invasive surgery [112,113].

Ploussard et al. investigated clinical outcomes in VHL patients treated with NSS. At 5- and 10-year intervals, the respective local recurrence rates were 45.6% and 83.7%, while the respective overall repeat surgery rates were 23.1% and 63.4%. The mean time to local recurrence was 53 months. The 10-year disease-specific survival rate was 93.8% and none of the NSS-treated patients progressed to CKD or developed metastatic disease [92].

### 4.2. Metastatic Disease

Currently, only scarce to moderate evidence guides therapeutic decision-making in the setting of metastatic VHL-related ccRCCs, and data are mostly extrapolated from the treatment approach to its sporadic counterpart. Metastatic RCCs are treated with target-therapy agents, mostly tyrosine-kinase inhibitors targeting VHL disease pathways, particularly the VEGF, PDGFR, and FGFR pathways. These agents include Semaxanib, Sunitinib, Pazopanib.

Complete radiologic and metabolic response of a metastatic RCC was reported after 11 administrations of Semaxanib, a tyrosine-kinase inhibitor that selectively targets VEGF [114].

Sunitinib is the most studied drug in VHL-related advanced RCC, and the main application is for palliative purposes, although complete remissions have been reported.

Several trials have demonstrated disease stability, partial response, and even complete regression [115] in metastatic RCCs treated with sunitinib [115,116,117,118,119,120,121]. Jonasch et al. documented a partial response rate of 33% in metastatic RCC patients treated with sunitinib, a better result than in hemangioblastomas. The authors speculated that the results could be explained by the greater expression of VEGFR2 in RCC cells [116].

Pazopanib is a 1st generation inhibitor of multiple tyrosine kinases (VEGFR-1/2/3, FGFR3, PDGFR α/β), approved for the palliative treatment of advanced RCC, seemingly with greater efficacy than sunitinib, albeit its use is supported by fewer and more inconsistent data [122,123].

Dovitinib is a multiple TKi; it is currently being studied for use in metastatic carcinoma, and the best response was found to be a stable disease for CNS hemangioblastomas [124].

Sorafenib is a multiple TKi; partial response in two patients was documented in patients with multiple small RCCs [125].

Although HIF2a inhibition has a strong biological rationale for the inhibition of an upstream abnormal target rather than one or more downstream effectors [38], to date, Belzutifan has not been evaluated in the metastatic stage of ccRCCs in VHL patients, thus other data are needed to support its use in this population. Clinical trials on Belzutifan in ccRCC, potentially also including VHL disease-ccRCC, are underway [126]. Interestingly, evidence is growing with regard to its use in advanced sporadic ccRCCs. A phase I/II trial on its use as monotherapy and an early analysis of a phase II trial on its combination with Cabozantinib both showed a favorable safety profile and promising antitumor activity [58,127].

## 5. MicroRNA Biomarkers of VHL-Associated Hereditary ccRCC

Even though the diagnosis of VHL syndrome is related to a well-known list of clinical and genetic features, as mentioned in the previous paragraph, the need for new molecular biomarkers able to predict the development and the aggressiveness of renal neoplasms in the VHL syndrome remains crucial in the oncological and nephrological panorama. In fact, one of the main problems related to VHL patients is represented by the multiple growths of ccRCC inside both kidneys, leading to multiple and consecutive surgical and radiological operations, when it is feasible [128]. As a result, VHL patients affected by ccRCC display an augmented risk of developing a moderate to an advanced stage of chronic kidney disease (CKD) due to the repetitive loss of nephrons mass [108], with a worse prognosis over time. Therefore, it would be fundamental for clinicians to better stratify VHL patients at risk of developing new ccRCC in order to avoid diagnostic delay leading to major surgical approaches such as radical nephrectomy in respect to conservative techniques such as nephron-sparing surgery [129]. In fact, the creation of a new molecular panel of circulating predictive biomarkers could help physicians to immediately highlight patients with a high predisposition of ccRCC development to follow a precise new medical algorithm able to detect and eliminate renal neoplasms at early stages with minimally invasive techniques, such as thermal or cryo-ablations, with the maximum saving of renal parenchyma over time [130]. In the molecular scenario, non-coding RNAs represent one of the most promising classes of predictive biomarkers in several types of malignancies and chronic conditions [131,132]. Non-coding RNAs are regulatory RNAs with no or little protein-coding potential [133]. However, in the last decade, several studies have highlighted their importance in the modulation of cell physiology and also cellular functions [134]. Among them, microRNAs are small non-coding single-stranded RNA molecules of 22 nucleotides in length that play a crucial role in the regulation of gene expression, highly involved in many functional processes [135]. Numerous studies both on solid and liquid biopsies have focused on microRNA expression profiles in sporadic ccRCC patients and have identified different miRNAs that act as oncogenes or tumor suppressors, regulating the main signaling pathways of ccRCC [136]. Moreover, different lines of evidence demonstrated that some panels of microRNAs could predict and determine the presence and aggressiveness of sporadic ccRCC, resulting in promising clinical biomarkers [135]. Surprisingly, only one report has so far studied the miRNA expression profile in VHL-associated hereditary ccRCC and compared that with the miRNA expression profile in sporadic ccRCC [137]. In this study, the authors characterized the mRNA and miRNA transcriptome (i) of several samples from multiple tumors occurring within the kidney of two patients affected with VHL disease and (ii) of 12 VHL-associated ccRCC, 22 sporadic ccRCC, and 17 normal adjacent tissues samples from patients with sporadic ccRCC [137]. They found that multiple kidney tumors in a patient affected with VHL disease show a very similar pattern of miRNAs ad mRNAs expression [137]. In addition, by using unsupervised hierarchical clustering of miRNA expression profiles, they were not able to discriminate between the two patients affected by VHL disease. However, the mRNA expression profile allows for the distinction between the two patients, maybe due to the different genetic background [137]. So, the VHL-associated hereditary ccRCC seems to be more homogeneous compared to the sporadic ccRCC. The high similarity in miRNA and mRNA expression indicated a similar molecular evolution of these synchronous tumors and suggested that the same molecular mechanisms underlie the pathogenesis of these hereditary tumors. As a matter of fact, reduced mutation burden and limited evidence of intra-tumor heterogeneity were detected in these tumors that exhibit complementarity of the evolutionary principles of contingency and convergence [138]. On the other hand, compared to normal reference samples, a total of 1377 genes and 51 miRNAs and a total of 1282 genes and 56 miRNAs were differentially expressed in the tumor samples of the two patients affected with VHL disease, respectively [137]. In addition, by using unsupervised hierarchical clustering analyses on the miRNA and mRNA expression profiles, the authors showed that tumor samples were well separated from the normal renal tissues. KEGG biological pathway analysis on dysregulated genes showed that three classes of pathways were overrepresented, and the most significant ones were similar in the two patients and were implicated in ‘immunity’ and ‘metabolism’ [137]. In addition, the authors found a total of 103 miRNAs differentially expressed in ccRCC samples compared to normal renal tissues. Sixty-eight are commonly dysregulated (12 upregulated and 56 downregulated) miRNAs in both VHL-associated and sporadic ccRCC. The authors identified also 18 differentially expressed miRNAs by directly comparing the VHL-associated and sporadic tumors (Table 2).

So, miRNA expression levels can distinguish VHL-associated tumors from sporadic ccRCC even though most differentially expressed miRNAs were similar between the two tumor groups [137]. Furthermore, by transcriptomic analysis, the authors showed that the mRNA expression profile was similar in VHL-associated and sporadic ccRCC. Among the dysregulated mRNAs in ccRCC samples compared to normal renal tissue samples, 2474 genes (959 up- and 1515 downregulated) were in common between VHL-associated and sporadic ccRCC, representing 71% and 81% of total dysregulated mRNAs in the two groups of samples, respectively [137]. So, even if VHL-associated and sporadic ccRCC are considered similar, more in-depth studies are needed to confirm this fact. The study of Gattolliat et al. showed similarities between the two entities, but also divergences in terms of miRNAs and mRNAs profiles helpful to distinguish and better characterize them [137]. One possible limitation of the above-mentioned study is represented by the fact that the relevant miRNA signatures were identified only in the tumor tissue of VHL patients, but not confirmed in the circulating plasma. From a precision medicine perspective, as described at the beginning of this paragraph, the use of a liquid biopsy instead of a tissue one could be fundamental for all VHL patients in order to avoid surgical or medical procedures (e.g., renal biopsy) which have the effect of damage or reduce the amount of nephron mass. Therefore, to find a promising tool for microRNAs in VHL patients, one should consider if the underlined tissue molecular signatures could reflect or not the circulating ones. However, different studies elucidated that tissue-derived microRNAs could be also completely different from the circulating ones, especially in the bloodstream where different organs can excrete different microRNAs and at different levels [139]. While new clinical trials will better investigate these aspects, a possible partial solution could be offered by the already studied circulating microRNAs in sporadic ccRCC, due to the overlap between the tumoral histology of sporadic and VHL ccRCC. As deeply described in a recent review of our group [136], different panels of circulating plasma and urine microRNAs in the ccRCC panorama have been investigated in both retrospective and prospective large studies, with promising results. Even though the absence of a reliable study in VHL patients concerning the evaluation of circulating microRNAs as predictive and prognostic biomarkers remains a gap to be filled, the confirmation or not of the already explored microRNAs in sporadic ccRCC in VHL patients could be a first step in the generation of a new molecular tool able to enter clinical practice.

## 6. Conclusions

Unlike research studies on the genetic bases of VHL disease, research studies on the transcriptome and non-coding RNA profiles on both liquid and solid biopsies are very scarce or absent in the context of VHL-associated hereditary ccRCC. The discovery of new biomarkers able to guide diagnosis, local or systemic therapy, and follow-up of VHL patients affected by ccRCC are urgent clinical needs. Given that non-coding RNA are emerging new molecular biomarkers for a plethora of diseases, including ccRCC, and could be promising therapeutic targets, preclinical and clinical research on the topic must be encouraged to improve the management of VHL patients.

## Figures and Tables

**Table 1:** Genetic alterations, biological pathways of RCC in VHL disease.

Genetic Alterations	Biological Pathways
3p loss	PI3K activation (possible role in conferring lethality), PI3K-AKT-mTOR (possible therapeutic target)
5q gain	DNA methylation pathway
VHL mutation	p53
VHL methylation	mRNA processing
PBRM1 mutation	TGFα, EGFR expression (cell proliferation and survival)
SETD2 mutation	PDGFB (angiogenesis, lymphangiogenesis)
BAP1 mutation	CXCR4, MMP2/9/14, lysyl oxidase, UPAR, MMP2 (tumoral cell invasion and metastases)
DNA repair defects	dysregulation of TWIST and activation of HGFR (EMT)
Defect in mitosis	VEGFA (angiogenesis, therapeutic target)
9p gain	
14q gain	
8q gain	
TCEB1 mutation	
Loss of segments of the small arm of chromosome 3 (the VHL gene-harboring 3p25-26, 3p12, 3p13-14.2, 3p21	

AKT = protein kinase B; BAP1 = BRCA1 associated protein-1; CXCR4 = C-X-C chemokine receptor type 4; EGFR = epidermal growth factor receptor; EMT = epithelial-to-mesenchymal transition; HGFR = hepatocyte growth factor receptor; MMP = matrix metalloproteinase; mRNA = messenger ribonucleic acid; mTOR = mammalian target of rapamycin; PBRM1 = protein polybromo-1; PDGFβ = platelet derived growth factor subunit β; PI3K = phosphatidylinositol 3-kinase; SETD2 = SET domain containing 2; TGFα = transforming growth factor α; TCEB1 = transcription elongation factor B polypeptide 1; TWIST = twist related protein; UPAR = urokinase-type plasminogen activator receptor; VEGFA = vascular endothelial growth factor A; VHL = Von Hippel-Lindau;.

**Table 2:** Dysregulated miRNAs between VHL-associated and sporadic ccRCC samples, adapted from ref. 136.

miRNA	Upregulated/ Downregulated	Fold-Change (VHL/Sporadic)	Raw *p*-Value	Adj *p*-Value
hsa-miR-489	Upregulated	2.267	0.0103	ns
hsa-miR-204	Upregulated	2.266	0.0078	ns
hsa-let-7f	Upregulated	1.946	0.0004	0.0123
hsa-miR-200b	Upregulated	1.914	0.0012	0.0216
hsa-let-7a	Upregulated	1.821	5.8836 × 10^−05^	0.0036
hsa-miR-200a	Upregulated	1.767	0.0035	0.0454
hsa-miR-146b-5p	Upregulated	1.684	0.0283	ns
hsa-miR-429	Upregulated	1.611	0.0066	ns
hsa-miR-26b	Upregulated	1.579	0.0004	0.0121
hsa-miR-28-5p	Upregulated	1.542	0.0006	0.0121
hsa-miR-122	Upregulated	1.527	0.0347	ns
hsa-miR-20a	Upregulated	1.521	0.0002	0.0092
hsa-miR-1274a	Downregulated	−1.58	0.0114	ns
hsa-miR-1260	Downregulated	−1.727	0.0027	0.0386
hsa-miR-886-3p	Downregulated	−1.764	0.0399	ns
hsa-miR-1308	Downregulated	−1.812	0.0136	ns
hsa-miR-494	Downregulated	−2.882	6.0369 × 10^−05^	0.0036
hsa-miR-923	Downregulated	−4.149	2.0833 × 10^−06^	0.0006

miRNA, microRNA; ns, not significant; VHL, von Hippel-Lindau.

**Table 3:** Summary of the clinical evidence on drugs or compounds evaluated in VHL disease ccRCC or with advanced ccRCCs.

Drug or Compound	Clinical Scenario	NCT or PMID	Phase
17AAG	VHL disease-related RCC	NCT00088374	2
Vandetanib	VHL disease-related RCC	NCT00566995	2
PT2385	VHL disease-related RCC	NCT03108066	2
Belzutifan as single agent	VHL disease-related tumor, including RCC; VHL disease-related RCC; Advanced ccRCCs, including sporadic and VHL-related tumors; Advanced solid tumors, including ccRCCs	NCT04924075; NCT03401788; NCT04846920; NCT02974738	2 2 1 1
Belzutifan plus Cabozantinib	Advanced ccRCCs	NCT03634540	2
Sunitinib	VHL disease-related tumor, including RCC; VHL disease-related tumor, including RCC Metastatic VHL-related RCC, case report; VHL disease-related tumor, including metastatic RCC Metastatic VHL disease-related RCC, retrospective analysis; VHL-disease-related tumor, including metastatic RCC, case series	NCT00330564; NCT01168440; 26881543; 22105611; 25391617; 24454008	2 2 / 2 / /
Pazopanib	VHL disease-related tumor, including RCC	NCT01436227	2
DFF332 as single agent or in combination with Everolimus or Spartalizumab plus Taminadenant	Tumors with HIF-stabilizing mutations (including also VHL disease-related ccRCC)	NCT04895748	1
Sorafenib	Recurrent stage T1 bilateral VHL-related RCC, case report	26425233	/
Dovitinib	VHL-related CHB, 33% of recruited patients had also RCC	NCT01266070	2
Semaxinib	Metastatic VHL-related RCC, case report	15271314	/

17AAG = 17-allylaminogeldanamycin; ccRCC = clear cell renal cell carcinoma; CHB = central nervous system hemangioblastoma; NCT = national clinical trial; PMID = PubMed identifier; PT2385 = 3-{[(1s)-2,2-Difluoro-1-Hydroxy-7-(Methylsulfonyl)-2,3-Dihydro-1h-Inden-4-Yl]oxy}-5-Fluorobenzonitrile; RCC = renal cell carcinoma; VHL = Von Hippel-Lindau.
